# Supplementary material for: Preadapted to adapt: underpinnings of adaptive plasticity revealed by the downy brome genome
Source: Commun Biol. 2023 Mar 27;6:326. doi: 10.1038/s42003-023-04620-9 (PMC10042881; doi:10.1038/s42003-023-04620-9)
Supplement: Supplementary file 2 — Supplemental Information [file 42003_2023_4620_MOESM2_ESM.docx]

**Supplementary Information**

**Supplementary Note 1**

**GWAS SNP Calling.** The SNP calling and filtering process resulted in 10522078 variants before filtering and 695263 SNPs after filtering by quality score > 30, and MAF > 0.05. After filtering out SNPs with more than two allelic states there were 691378 SNPs Remaining. Once sites with 5 heterozygotes or more were removed 119579 SNP remained.

**Linkage-disequilibrium.** The LD_90,1/2_ occurred at 162023 bp. From the asymptotic decay model, the 90^th^ percentile of LD started at 0.719 when the physical distance was zero bp decaying halfway to the asymptotic limit (r^2^ = 0.17) at 0.36 within 162023 bp (Figure S2). The natural logarithm of the rate constant estimate was -11.9 for the asymptotic decay model.

***Table S1.*** Genome draft assembly statistics for the OmniC assembly.

| **Statistic** | **OMNI-C** |
| --- | --- |
| Length of Assembly (mb) | 2,482.01 |
| Contig N50^[[1]](#endnote-1)^ (kb) | 19,386.61 |
| Scaffold N50 (mb) | 357.431 |
| Scaffold L50^[[2]](#endnote-2)^ | 4 |
| Scaffold N90^[[3]](#endnote-3)^ (mb) | 276.77 |
| Scaffold L90^[[4]](#endnote-4)^ | 7 |
| Number of Gaps | 259 |
| Single-gene BUSCO^[[5]](#endnote-5)^ | 1455 (91.1.2%) |
| Duplicated BUSCO | 26 (1.6%) |
| Fragmented BUSCO | 6 (0.4%) |
| Missing BUSCO | 127 (7.9%) |

***Table S2.*** Genome draft assembly genome feature and repeat information.

| **Class** | **Repeat** | **bp (number of genes)** | **%masked** | **%genome** |
| --- | --- | --- | --- | --- |
| Gene |  | 49901610 (1298 genes) |  | 2.01 |
| DNA |  | 3141552 | 0.15 | 0.13 |
|  | CMC-EnSpm | 89379930 | 4.33 | 3.60 |
|  | MULE-MuDR | 4819353 | 0.23 | 0.19 |
|  | MuLE-MuDR | 3635933 | 0.18 | 0.15 |
|  | PIF-Harbinger | 10180198 | 0.49 | 0.41 |
|  | TcMar-Stowaway | 6720836 | 0.33 | 0.27 |
|  | hAT-Ac | 1461846 | 0.07 | 0.06 |
|  | hAT-Tag1 | 1127382 | 0.05 | 0.05 |
|  | hAT-Tip100 | 362982 | 0.02 | 0.01 |
| LINE | CR1 | 1004101 | 0.05 | 0.04 |
|  | Jockey | 701674 | 0.03 | 0.03 |
|  | L1 | 33384872 | 1.62 | 1.35 |
|  | RTE-BovB | 2492881 | 0.12 | 0.10 |
| LTR |  | 2360344 | 0.11 | 0.10 |
|  | Caulimovirus | 644197 | 0.03 | 0.03 |
|  | Copia | 266297378 | 12.90 | 10.73 |
|  | Gypsy | 1447726437 | 70.14 | 58.33 |
| RC | Helitron | 1289186 | 0.06 | 0.05 |
| SINE | L1 | 1524560 | 0.07 | 0.06 |
|  | tRNA | 1347247 | 0.07 | 0.05 |
| Unknown |  | 163537694 | 7.92 | 6.59 |
| Total interspersed | | 2043140583 | 98.99 | 82.32 |

***Figure S1*.** Histograms for phenology traits and height for each trait. For each trait there are histograms for each of the sampling sources for the association panel. Brown represents GRIN genotypes, blue represents Washington genotypes, and yellow represents Montana genotypes. Panels are labeled by respective trait.


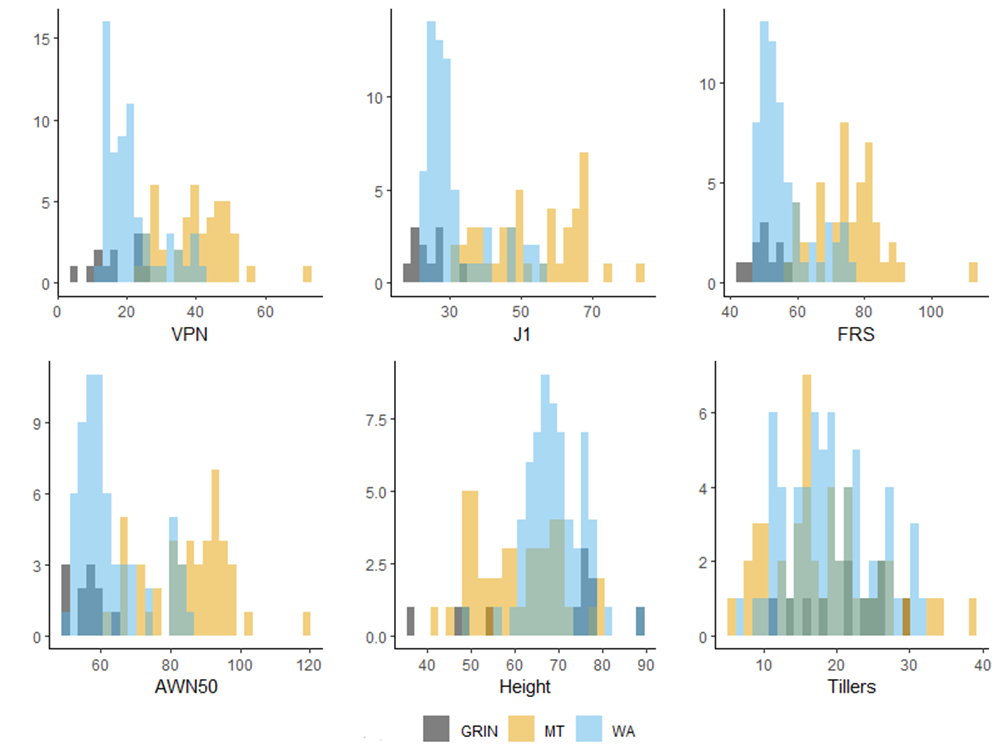


***Figure S2.*** Asymptotic decay of linkage disequilibrium (LD) as a function of distance in *Bromus tectorum*. Green line is the asymptotic decay curve of best fit for the 90^th^ percentile of LD values.


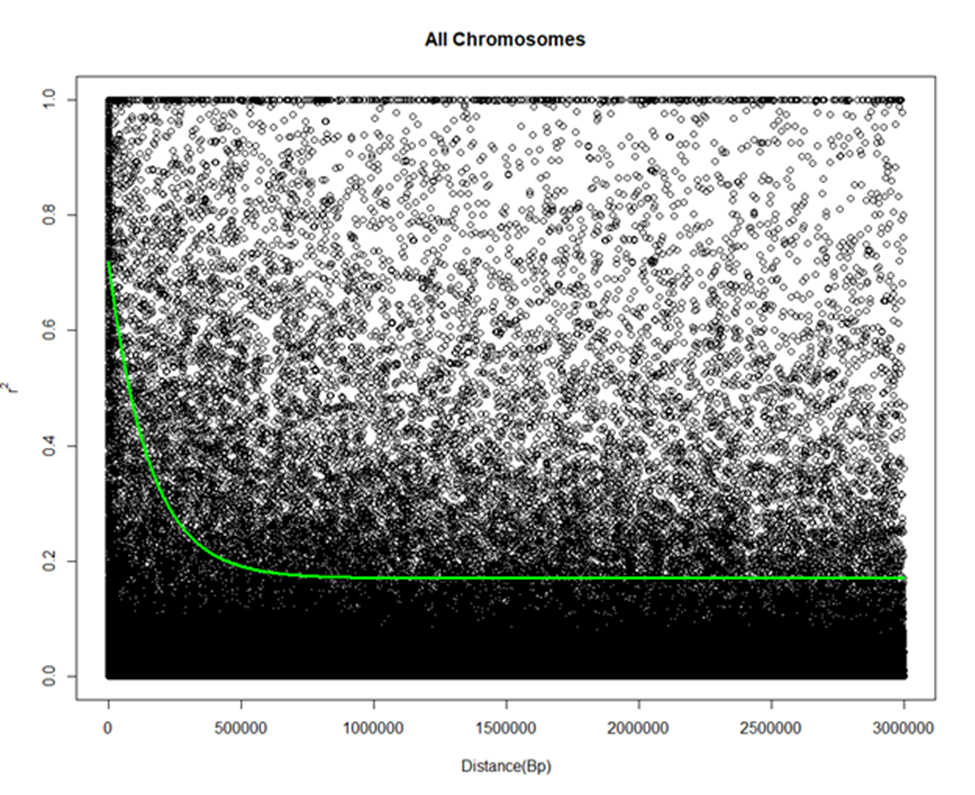


1. Length of shortest contig at 50% of the total genome length [↑](#endnote-ref-1)
2. Minimum number of scaffold to explain 50% of the total genome length [↑](#endnote-ref-2)
3. Length of shortest contig at 90% of the total genome length [↑](#endnote-ref-3)
4. Minimum number of scaffold to explain 90% of the total genome length [↑](#endnote-ref-4)
5. Benchmarking universal single-copy orthologs [↑](#endnote-ref-5)
