# Supplementary material for: Preadapted to adapt: underpinnings of adaptive plasticity revealed by the downy brome genome
Source: Commun Biol. 2023 Mar 27;6:326. doi: 10.1038/s42003-023-04620-9 (PMC10042881; doi:10.1038/s42003-023-04620-9)
Supplement: Supplementary file 3 — Description of Additional Supplementary Data [file 42003_2023_4620_MOESM3_ESM.docx]

**Description of Additional Supplementary Files**

**File name:** Supplementary Data 1

**Description:** Names, location and sources for genotypes re-sequenced in study.

**File name:** Supplementary Data 2

**Description:** Best Linear Unbiased Estimates (BLUEs) for days to first joint (J1), days to 50% ripe seed (AWN50), days to first ripe seed (FRS), days to first visible panicle (VPN), number of tillers, and height (cm). All traits except height are log transformed because of the log-transformation involved with the generalized linear mixed model with a Poisson distribution to account for count variables. Height is untransformed.
