# Supplementary material for: Preadapted to adapt: underpinnings of adaptive plasticity revealed by the downy brome genome
Source: Commun Biol. 2023 Mar 27;6:326. doi: 10.1038/s42003-023-04620-9 (PMC10042881; doi:10.1038/s42003-023-04620-9)
Supplement: Supplementary file 6 — Nature Reporting Summary [file 42003_2023_4620_MOESM6_ESM.pdf]

nature portfolio

Corresponding author(s): Ian C. Burke

Last updated by author(s): 2/8/2023

Reporting Summary

Nature Portfolio wishes to improve the reproducibility of the work that we publish. This form provides structure for consistency and transparency in reporting. For further information on Nature Portfolio policies, see our Editorial Policies and the Editorial Policy Checklist.

Statistics

For all statistical analyses, confirm that the following items are present in the figure legend, table legend, main text, or Methods section.

n/a	Confirmed	
☐	☒	The exact sample size (*n*) for each experimental group/condition, given as a discrete number and unit of measurement
☒	☐	A statement on whether measurements were taken from distinct samples or whether the same sample was measured repeatedly
☐	☒	The statistical test(s) used AND whether they are one- or two-sided<br>*Only common tests should be described solely by name; describe more complex techniques in the Methods section.*
☐	☒	A description of all covariates tested
☐	☒	A description of any assumptions or corrections, such as tests of normality and adjustment for multiple comparisons
☐	☒	A full description of the statistical parameters including central tendency (e.g. means) or other basic estimates (e.g. regression coefficient) AND variation (e.g. standard deviation) or associated estimates of uncertainty (e.g. confidence intervals)
☐	☒	For null hypothesis testing, the test statistic (e.g. *F*, *t*, *r*) with confidence intervals, effect sizes, degrees of freedom and *P* value noted<br>*Give P values as exact values whenever suitable.*
☒	☐	For Bayesian analysis, information on the choice of priors and Markov chain Monte Carlo settings
☒	☐	For hierarchical and complex designs, identification of the appropriate level for tests and full reporting of outcomes
☐	☒	Estimates of effect sizes (e.g. Cohen's *d*, Pearson's *r*), indicating how they were calculated

Our web collection on statistics for biologists contains articles on many of the points above.

Software and code

Policy information about availability of computer code

Data collection	No computer code was used to collect data
Data analysis	Python code for automating the Debian Unix bioinformatics tools and R code for the analysis are available on figshare

For manuscripts utilizing custom algorithms or software that are central to the research but not yet described in published literature, software must be made available to editors and reviewers. We strongly encourage code deposition in a community repository (e.g. GitHub). See the Nature Portfolio guidelines for submitting code & software for further information.

Data

Policy information about availability of data

All manuscripts must include a data availability statement. This statement should provide the following information, where applicable:

- Accession codes, unique identifiers, or web links for publicly available datasets
- A description of any restrictions on data availability
- For clinical datasets or third party data, please ensure that the statement adheres to our policy

The raw sequences used for the B. tectorum genome assembly are deposited in the National Center for Biotechnology Information (NCBI) Sequence Read Archive database under the BioProject PRJNA728981 with the following accession numbers: SRR14498212–SRR14498217 (PacBio reads), SRR14578284–SRR14578290 (Hi-C reads), SRR14578282 (Transcriptome) and SRR14498209–SRR14498211 (Polishing short reads). The raw reads for the resequencing panel are found in BioProject PRJNA728981 with the following NCBI accession numbers: SRR15308470–SRR15308851 (resequencing panel). Genome browsing and bulk data downloads,

including annotations and BLAST analysis of the final proximity-guided assemblies are available at CoGe (https://genomevolution.org/coge/) with genome ID:id64356.

Code Availability

The custom scripts, phenotype data and SNP data have been deposited in figshare (https://doi.org/10.6084/m9.figshare.c.6419786.v1)

Human research participants

Policy information about studies involving human research participants and Sex and Gender in Research.

Reporting on sex and gender	No human participants and downy brome has complete flowers thus does not have multiple sexes.
Population characteristics	There were no human participants
Recruitment	There were no human participants
Ethics oversight	There were no human participants

Note that full information on the approval of the study protocol must also be provided in the manuscript.

Field-specific reporting

Please select the one below that is the best fit for your research. If you are not sure, read the appropriate sections before making your selection.

☐ Life sciences ☐ Behavioural & social sciences ☒ Ecological, evolutionary & environmental sciences

For a reference copy of the document with all sections, see nature.com/documents/nr-reporting-summary-flat.pdf

Ecological, evolutionary & environmental sciences study design

All studies must disclose on these points even when the disclosure is negative.

Study description	Genotypes of Downy brome were replicated as inbred lines (due to almost 100% selfing). Two greenhouses were used with 3 replicates of each genotype in each greenhouse. Thus there were two environments and 6 total replicates for each genotype.
Research sample	Genotypes were opportunistically sampled based on what was available and grew correctly. Many genotypes from Lisa Rew and some of the genotypes from Washington had smut and were not able to be increased in seed. Only genotypes where smut was not present were used. Also some of the Washington genotypes had extreme dormancy and wouldnt germinate thus excluding them from the study. We selected 200 genotypes because the sequencing company LGC would give us a better deal at two 96 well plates. For genotypes, the number of samples from each location is not critical for the GWAS because GWAS methods include PCA and kinship thus controlling for the population structure.
Sampling strategy	Replicating the each genotype a total of 6 times (3 reps in each green house) was done because we did not know the heritability of the traits previously so we decided to error on the side of having more replicates then necessary.
Data collection	Data was collected each day by Samuel Revolinski. Eat day each plant would be checked to see if it had reached growth stages, when growth stages (reproductive phenology traits) were met a plastic stake with the growth stage reached and the corresponding date was placed into the pot the plant was in. At the end of the study, Samuel Revolinski counted the number of tillers on each plant and the height of the tallest panicle (using meter stick).
Timing and spatial scale	Reproductive phenology data was collected on a daily basis from 7/24/2022 to 12/29/2020. The expirements took place in two greenhouses simultaneously.
Data exclusions	For genotypes grown in the experiment no data was excluded.
Reproducibility	The reliability (or broad sense heritability) measure showed that the experiments were highly repeatable across greenhouses in respect to the genotype.
Randomization	Genotypes although collected in groups were not organized into groups to test hypothesis based on those groups. Each block contained all of the genotypes randomized within the block. Each block was randomized individually so that each block has a unique randomization of the genotypes.
Blinding	Blinding was not relevant to the study because there was no placebo and control. Additionally, the measurements and phenotyes taken are highly specific in that certain criteria must be met, reducing the risk of Bias.

Did the study involve field work? ☐ Yes ☒ No

Reporting for specific materials, systems and methods

We require information from authors about some types of materials, experimental systems and methods used in many studies. Here, indicate whether each material, system or method listed is relevant to your study. If you are not sure if a list item applies to your research, read the appropriate section before selecting a response.

Materials & experimental systems

n/a	Involved in the study
☒	☐ Antibodies
☒	☐ Eukaryotic cell lines
☒	☐ Palaeontology and archaeology
☐	☒ Animals and other organisms
☒	☐ Clinical data
☒	☐ Dual use research of concern

Methods

n/a	Involved in the study
☒	☐ ChIP-seq
☒	☐ Flow cytometry
☒	☐ MRI-based neuroimaging

Animals and other research organisms

Policy information about studies involving animals; ARRIVE guidelines recommended for reporting animal research, and Sex and Gender in Research

Laboratory animals	Study did not involve animals
Wild animals	study did not involve animals
Reporting on sex	bromus tectorum has perfect hermaphroditic flowers thus there are not multiple sexes of bromus tectorum.
Field-collected samples	The original field samples that were used to create the set of genotypes were grown in uniform conditions in the greenhouse for one generation to increase seed and minimize maternal effects before starting the trials. The conditions they were grown in for the single generation is similar to the study except only one replicate of each genotype was grown for seed.
Ethics oversight	No ethical approval was needed because bromus tectorum is an invasive plant species and the samples used in this study was performed using previously collected materials.

Note that full information on the approval of the study protocol must also be provided in the manuscript.
